# Supplementary material for: A Honey Bee In-and-Out Counting Method Based on Multiple Object Tracking Algorithm
Source: Insects. 2024 Dec 6;15(12):974. doi: 10.3390/insects15120974 (PMC11677362; doi:10.3390/insects15120974)
Supplement: Supplementary file 1 [file insects-15-00974-s001.zip › insects-3300542-supplementary.pdf]

Table S1. Parameters of the smart hive.

Contents	Parameters
Hive design	Langstroth
Hive Size (Inside)	510mm×410mm×335mm
Entrance Size	60mm×7mm
Power Supply	PoE
Data Transmission	WiFi
fps	30
Image Quality	720P
Power Dissipation	<15W
Waterproof Gradation	IP65
Operation Temperature	-10~+55℃
Operation Humidity	20 ~ 90%RH

Table S2. Details of MOT-BEE dataset.

Num.	Frame rate (FPS)	Number of frames	Number of ground truth target boxes
1	30	902	4212
2	30	596	3766
3	30	934	1728
4	30	863	1236
5	30	1181	12047
6	30	450	1327
7	30	604	3832
8	30	264	4803

Table S3. Details of IOC-BEE dataset.

Num.	Frame rate (FPS)	Environment	Number of frames	Manual incoming counts	Manual outgoing counts
1	30	Normal light intensity	901	21	17
2	30	Normal light intensity	1942	23	26
3	30	Normal light	1812	22	26

		intensity			
4	30	Normal light intensity	2145	34	28
5	30	High light intensity	1822	68	60
6	30	High light intensity	2745	74	62
7	30	Low light intensity	3982	14	14
8	30	Low light intensity	1795	4	5

Table S4. Details of MOT algorithms.

Algorithm	Tracking method	Improvement points
BotSORT	KF	Advantages of motion and appearance information were combined, along with camera-motion compensation, and a more accurate Kalman filter state vector.
StrongSORT	KF	An appearance-free link model (AFLink) is used to perform global association without appearance information. Gaussian-smoothed interpolation (GSI) based on Gaussian process regression is used to relieve missing detection.
ByteTRACK	KF	An association method named BYTE is introduced. BYTE can associate almost every detection box. Low-scoring detection boxes and unpaired tracklets were used to retrieve objects from the low-scoring detection boxes and filter out background elements.
OC-SORT	KF	A novel Observation-centric Re-Update (ORU) module is added to address accumulated errors during untracked periods. An additional observation-centric approach is added to incorporate the directional consistency of the trajectory into the Observation-Centric Momentum (OCM) in the associated cost matrix.
Deep OC-SORT	KF	Deep OC-SORT is built upon a modified version of OC-SORT. Dynamic Appearance (DA) and Adaptive Weighting (AW) techniques are implemented.
